# Performance of a HeartLogic^TM^ Based Care Path in the Management of a Real-World Chronic Heart Failure Population

**DOI:** 10.3389/fcvm.2022.883873

**Published:** 2022-05-06

**Authors:** Michelle Feijen, Anastasia D. Egorova, Roderick W. Treskes, Bart J. A. Mertens, J. Wouter Jukema, Martin J. Schalij, Saskia L. M. A. Beeres

**Affiliations:** ^1^Department of Cardiology, Leiden University Medical Center, Leiden, Netherlands; ^2^Department of Biomedical Data Sciences, Leiden University Medical Center, Leiden, Netherlands

**Keywords:** HeartLogic^TM^, chronic heart failure, multisensory remote monitoring, heart failure admissions, CIED

## Abstract

**Aim:**

Early detection of impending fluid retention and timely adjustment of (medical) therapy can prevent heart failure related hospitalizations. The multisensory cardiac implantable electronic device (CIED) based algorithm HeartLogic^TM^ aims to alert in case of impending fluid retention. The aim of the current analysis is to evaluate the performance of the HeartLogic^TM^ guided heart failure care path in a real-world heart failure population and to investigate whether the height of the index and the duration of the alert state are indicative of the degree of fluid retention.

**Methods:**

Consecutive adult heart failure patients with a CIED and an activated HeartLogic^TM^ algorithm were eligible for inclusion. Patients were followed up according to the hospital's heart failure care path. The device technician reviewed alerts for a technical CIED checkup. Afterwards, the heart failure nurse contacted the patient to identify impending fluid retention. An alert was either true positive or false positive. Without an alert a patient was true negative or false negative.

**Results:**

Among 107 patients, [82 male, 70 (IQR 60–77) years, left ventricular ejection fraction 37 ± 11%] 130 HeartLogic^TM^ alerts were available for analysis. Median follow up was 14 months [IQR 8–23]. The sensitivity to detect impending fluid retention was 79%, the specificity 88%. The positive predictive was value 71% and the negative predictive value 91%. The unexplained alert rate was 0.23 alerts/patient year and the false negative rate 0.17 alerts/patient year. True positive alerts [42 days (IQR 28–63)] lasted longer than false positive alerts [28 days (IQR 21–44)], *p* = 0.02. The maximal HeartLogic^TM^ index was higher in true positive alerts [26 (IQR 21–34)] compared to false positive alerts [19 (IQR 17–24)], *p* < 0.01. Patients with higher HeartLogic^TM^ indexes required more intense treatment (index height in outpatient setting 25 [IQR 20–32], day clinic treatment 28 [IQR 24–36] and hospitalized patients 45 [IQR 35–58], respectively), *p* < 0.01.

**Conclusion:**

The CIED-based HeartLogic^TM^ algorithm facilitates early detection of impending fluid retention and thereby enables clinical action to prevent this at early stage. The current analysis illustrates that higher and persistent alerts are indicative for true positive alerts and higher index values are indicative for more severe fluid retention.

## Introduction

Heart failure results in substantial mortality and morbidity and impairs quality of life (1–3). Hospitalizations are a marker of poor prognosis and pose a significant burden on patients and healthcare resources (1–5). Heart failure related care accounts for 1–2% of the healthcare budget of developed nations and projections show an expected 127% increase in heart failure costs by 2030 (6). The majority of these expenses are related to hospital admissions for decompensated heart failure (7). Early detection of fluid retention, even before the onset of symptoms, allows timely management in the outpatient clinic and may prevent hospitalizations.

Cardiac implantable electronic devices (CIEDs) reduce the risk of sudden arrhythmic death in heart failure patients with a reduced ejection fraction (3). However, CIEDs also allow for continuous monitoring of a range of physiological parameters through various sensors. The recently developed CIED based algorithm HeartLogic^TM^ (Boston Scientific St. Paul, United States) uses five sensors to detect impending fluid retention (8, 9). These five sensors assess the first and the third heart sounds (S1 and S3, respectively) and the S3/S1ratio, respiration rate, intrathoracic impedance, night heart rate and physical activity (9). All parameters are collected automatically and computed into the HeartLogic^TM^ index. The MultiSENSE validation study, that enrolled 900 heart failure patients with cardiac resynchronization therapy defibrillators, demonstrated that HeartLogic^TM^ can identify time-intervals when patients are at increased risk of worsening heart failure. When the HeartLogic^TM^ index nominal value of 16 was used to trigger an alert episode, the algorithm was able to detect 70% of impeding heart failure events with a median of 34 warning days before the clinical event. Apart from the MultiSENSE study (and several *post hoc* analyses from this study), data on the performance of the HeartLogic^TM^ algorithm are scarce. A few studies showed that patients in alert status have an increased risk of congestion as compared to patients not in alert status (10–13). HeartLogic^TM^ alerts are frequently actionable and clinical action seemed to lower the decompensated heart failure event rate compared to a wait and see strategy (10, 14). However, the definition of clinical action is not uniform and alert-based management protocols are not available. Furthermore, the positive and negative predictive values of an HeartLogic^TM^ alert have not yet been validated and the clinical relevance of the height of the index and the duration of the alert remains to be investigated. Accordingly, the aim of the current study is to evaluate the performance of a HeartLogic^TM^ guided care path in a real-world heart failure population and to investigate whether the height of the index and the duration of the alert state are indicative of the degree of fluid retention.

## Methods

### Study Population

All adult heart failure patients with a CIED implanted (de novo implants and pulse generator exchanges) from 01-01-2018 onwards and an active and calibrated HeartLogic^TM^ algorithm who were under follow-up at the Leiden University Medical Center until 01-05-2021, the Netherlands, were eligible for inclusion. Basic Dutch or English-speaking skills were necessary for inclusion. Patients with a Left Ventricular Assist Device (LVAD) or unwillingness to comply to the HeartLogic^TM^ heart failure care path were excluded from analysis.

### Data Collection

Demographic and clinical data were collected from the hospital patient information systems (EPD-Vision Leiden, the Netherlands and HiX Chipsoft Amsterdam, the Netherlands). Demographic characteristics included among others: age, gender, type of CIED, etiology of heart disease, left ventricular ejection fraction, co-morbidities and medication. The HeartLogic^TM^ index, parameters and trends were collected from the LATITUDE^TM^ platform (Boston Scientific St. Paul, The United States) and were documented (weekly) in a consecutive manner.

**Figure 1:**
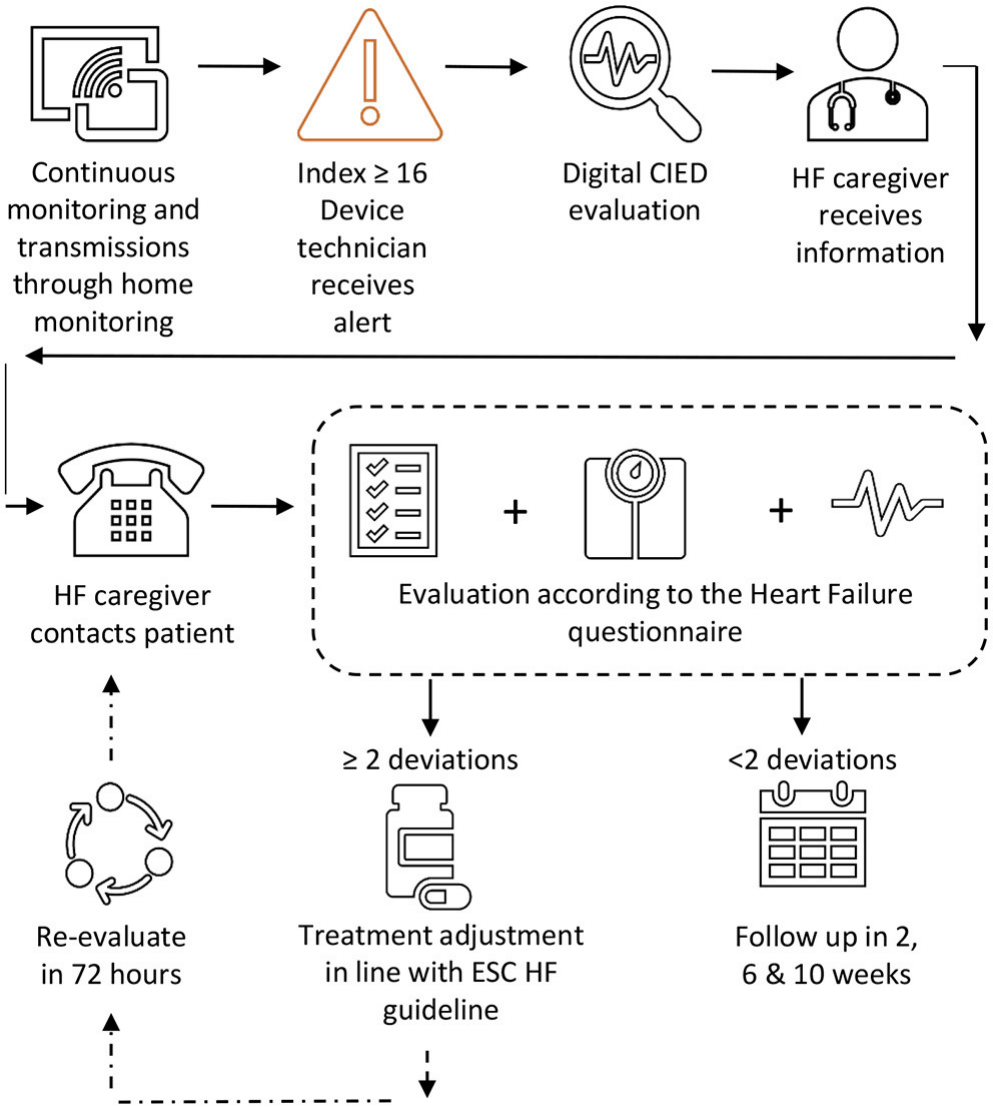
Heart failure care path.

Patients underwent systematic clinical follow-up according to the HeartLogic^TM^ guided heart failure care path, as previously described by Treskes et al. (15) (Figure 1). In brief, if the index surpassed the preset threshold of 16, an alert was automatically sent to the CIED team by the LATITUDE^TM^ platform. Trained device technicians first reviewed the alert for CIED related technical issues (e.g. deviations in lead threshold and impedance auto-measurements) and/or arrythmias. Thereafter, the alert and the CIED specific information was forwarded to the heart failure team. Subsequently, the heart failure nurses contacted the patient by phone for a digital evaluation within 72 h after the index surpassed the threshold. The nurse accessed the patient for early signs and symptoms of fluid retention. To this aid, a dedicated heart failure questionnaire, including weight and blood pressure measurements, was structurally used (Supplementary Figure 1).

If the evaluation revealed 2 or more criteria suggestive of fluid retention on top of the HeartLogic^TM^ alert, the alert was considered to be true positive. The severity of fluid retention and patient's symptoms determined the therapeutic course of action. As per protocol, lifestyle modification advice to reduce fluid and salt intake were given to all patients and a re-evaluation was scheduled after 2 weeks. In case of moderate congestion, the oral diuretic dose was doubled for 3 days and an extra evaluation was scheduled after 72 h. When congestion symptoms appeared severe, a single shot of intravenous (IV) diuretics was administrated during a day visit. If the effect of the above was insufficient, the patient was hospitalized. The treating cardiologist made the final decision regarding the given treatment. In case of clinically relevant (atrial) arrythmias, overpacing or cardioversion were planned as deemed necessary and possible by the treating cardiologist. When a primarily not heart failure related diagnosis was suspected, the patient was referred to the general practitioner for further investigation.

Alternatively, if the digital evaluation revealed <2 criteria suggestive of fluid retention (apart from the HeartLogic^TM^ alert), a digital re-evaluation of the HeartLogic^TM^ index and the clinical status was planned 2 weeks later. Patients without signs and symptoms of fluid retention were contacted digitally 2, 6, and 10 weeks after the initial index alert for a complete follow-up. An alert was considered false positive if during the 10-week follow-up period the digital evaluation did not reveal 2 or more symptoms or signs of heart failure and the patient did not present with worsening heart failure.

### Alerts

An alert was deemed true positive if at least two criteria (i.e., signs and symptoms of fluid retention) were met according to the heart failure questionnaire. Alerts with zero or maximum one criterium on the heart failure questionnaire were categorized as false positive if no clinical congestion occurred after completing the 10-week episode specific follow-up according to the heart failure care path. The index was truly negative, if a patient did not surpass the threshold and no signs or symptoms of fluid retention were reported. Finally, signs and symptoms of fluid retention according to the heart failure questionnaire without a preceding HeartLogic^TM^ alert were defined as false negative alerts.

### Outcome

The primary outcome of the study was the performance of the HeartLogic^TM^ algorithm for the prediction of fluid retention in ambulant chronic heart failure patients by assessing the sensitivity, specificity and positive and negative predictive values. Additional analysis was performed to evaluate whether the height of the index and the duration of the alert state were indicative of the degree of fluid retention.

### Statistical Analysis

Normally distributed data are reported as mean ± standard deviation (SD) and non-normally distributed data as median with interquartile range [IQR1 –IQR3], unless specifically stated otherwise. Normality was tested with use of the Kolmogorov-Smirnov and the Shapiro-Wilk tests. Logistic regression with random patient effects to account for repeated within patient observations were used to assess the performance of HeartLogic^TM^ alert. Sensitivity and specificity, as well as predictive values were determined based on logistic regression with generalized linear mixed models. Linear mixed-effect models to adjust for the repeated measures were used to asses differences in maximal alert height and alert duration. The linear mixed effect models were adjusted for within patient observations with random intercept per patient and either treatment or alert status as fixed effects. A *p*-value < 0.05 was considered significant. Statistical analysis was performed with IBM SPSS statistics (version 25).

### Ethics Statement

The study was conducted in accordance with the Declaration of Helsinki, applicable local laws and regulations and the European directive for data protection (General Data Protection Regulation). The local ethical committee approved the study protocol (G21.103) and each patient provided informed consent for participation in the study.

## Results

### Study Population

In total, 112 patients with a functional HeartLogic^TM^ algorithm on their CIED were assessed for eligibility. Of these, 5 were excluded from analysis: 3 patients had an LVAD and 2 patients withdrew consent from the structured care path follow-up. Accordingly, the study population comprised of 107 patients. As shown in Table 1, 82 patients (77%) were male, median age was 70 years [IQR 60–77], the etiology of heart failure was ischemic in 50 patients (47%) and median left ventricular ejection fraction was 37 ± 11%. At inclusion, 72 patients (67%) had a cardiac resynchronization therapy device with defibrillator function (CRT-D), and the remaining 35 patients (33%) had a single or double chamber internal cardioverter defibrillator (ICD). Median follow-up was 14 months [IQR 8–23] and the total follow-up comprised 148 patient years. During follow-up, 3 patients died: 1 due to end stage heart failure and 2 deaths were not heart failure related.

**Table 1:** Baseline characteristics of the patients included for analysis (*N* = 107).

	***N* = 107**
Age in years, median [IQR]	70 [60–77]
Male, *n* (%)	82 (77)
Time since HF diagnosis in years, median [IQR]	12 [4–16]
BMI in kg/m^2^, median [IQR]	27 [24–30]
LVEF in %, (SD)	37 ± 11
Reduced LVEF (≤ 40%), *n* (%)	68 (63)
Mildly reduced LVEF (41–49%), *n* (%)	25 (23)
Preserved LVEF, (≥ 50%), *n* (%)	14 (13)
NYHA class, *n* (%)	
I	25 (23)
II	52 (49)
III	25 (23)
IV	4 (5)
**Etiology**	
Ischemic, *n* (%)	50 (47)
Non -ischemic, *n* (%)	57 (53)
**Device**	
CRT, *n* (%)	72 (67)
Percentage biventricular pacing, median [IQR]	99 [96–100]
First CRT implant, *n* (%)	33 (31)
DDD/VVI ICD, *n* (%)	35 (33)
**Cardiac history**	
CABG, *n* (%)	19 (18)
Valve Surgery, *n* (%)	17 (16)
**Co-morbidities**	
Atrial Fibrillation, *n* (%)	43 (40)
Paroxysmal/persistent, *n* (%)	31 (72)
Permanent, *n* (%)	12 (28)
Hypertension, *n* (%)	50 (47)
COPD, *n* (%)	7 (7)
Diabetes Mellitus, *n* (%)	14 (13)
Ischemic CVA/TIA, *n* (%)	7 (7)
Chronic kidney disease	
Stage 1 (>90 mL/min), *n* (%)	13 (12)
Stage 2 (60–89 mL/min), *n* (%)	49 (46)
Stage 3 A (45–59 mL/min), *n* (%)	20 (19)
Stage 3 B (30–44 mL/min), *n* (%)	15 (14)
Stage 4 (15 −29 mL/min), *n* (%)	10 (9)
**Laboratory findings**	
NT-ProBNP ng/L, median [IQR]	870 [314 −3215]
Hemoglobin mmol/L, median [IQR]	8.5 [7.7- 9.0]
**Medical therapy**	
Beta-blocker, *n* (%)	94 (88)
ACE-I/ARB/ARNI, *n* (%)	96 (90)
ACE-I, *n* (%)	55 (52)
ARNI, *n* (%)	15 (14)
ARB, *n* (%)	26 (24)
MRA, *n* (%)	58 (54)
Diuretics, *n* (%)	75 (70)
Loop, *n* (%)	66 (62)
Thiazides, *n* (%)	9 (8)
Ivabradine, *n* (%)	2 (2)
Digoxin, *n* (%)	5 (5)

*ACE-I, angiotensin-converting enzyme inhibitor; ARB, angiotensin II receptor blockers; ARNI, angiotensin receptor- neprilysin inhibitor; BSA, body surface area; BMI, body mass index; CABG, coronary artery bypass graft; COPD, chronic obstructive pulmonary disease; CRT-D, cardiac resynchronization therapy with defibrillator; CVA, cerebrovascular accident; eGFR, estimated glomerular filtration rate; ICD, implantable cardioverter defibrillator; LVEF, left ventricular ejection fraction; MRA, mineral corticoid inhibitor; NT-Pro BNP, n-terminal pro B-type natriuretic peptide; NYHA, new york heart association class*.

### HeartLogic^TM^ Alerts

**Figure 2:**
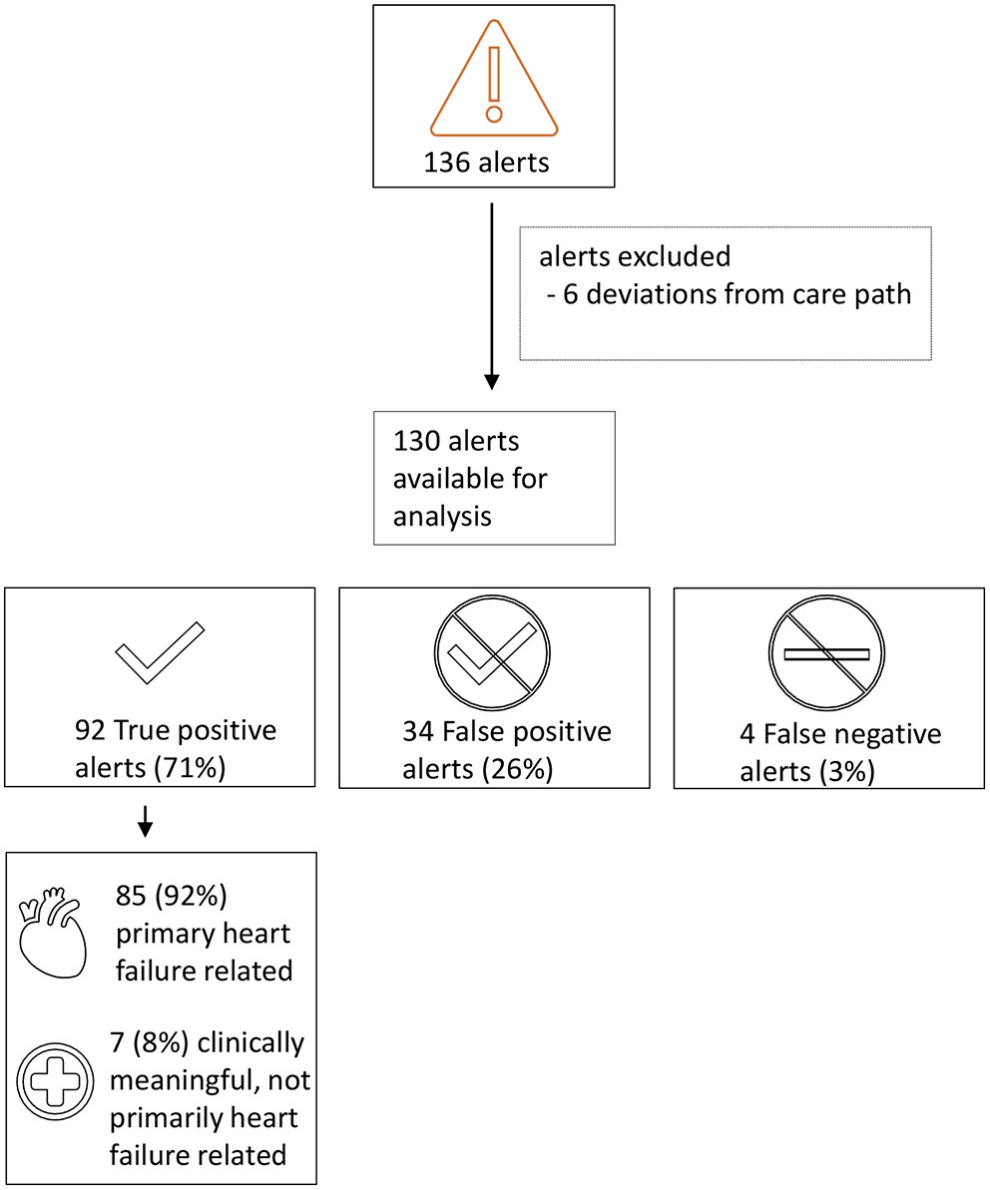
Flowchart of the HeartLogic^TM^ alerts during follow-up.

In 107 patients, 136 HeartLogic^TM^ alerts episodes occurred during the study period. Of these, 6 were excluded from analysis due to deviations from the heart failure care path in the way the alerts were handled. Accordingly, 130 alerts that occurred in 54 patients were available for analysis (Figure 2). Most patients experienced 1 alert episode during follow-up (*n* = 27, 50%), 16 (30%) patients had 2 or 3 alert episodes, 6 (11%) patients had 4 or 5 alert episodes and 5 (9%) patients experienced 6 or more alert episodes (maximum of 9 alert episodes). The remaining 53 patients had no alerts during follow-up. On average, each patient experienced 0.88 alert episodes/patient year with a median duration of 35 days per alert [IQR 28–56].

### Clinical Performance of the HeartLogic^TM^ Alert

According to the criteria defined in the heart failure care path, 92 alerts (71%) were true positive for fluid retention (Figure 2). Of interest, 7 of these true positive alerts were clinically relevant, but not primary heart failure related (e.g., fluid retention triggered by a pneumonia or significant anemia). Of the remaining alerts, 34 (26%) alerts were false positive. The unexplained alert rate (UAR) was 0.23 alerts per patient year. Four alerts (3%) were issued after the patients already experienced signs and symptoms of fluid retention and therefore were allocated as false negative for timely detection of impending fluid retention. Furthermore, 19 relatively short (median 4 days [IQR 3–17]) episodes of mild fluid retention not requiring hospitalization were not detected by the HeartLogic^TM^ algorithm. The false negative rate was 0.17 events per patient year.

Based on these 130 alerts, logistic regression with generalized linear mixed models estimated that the sensitivity of the HeartLogic^TM^ algorithm based care path to detect early signs of fluid retention was 79% (CI 0.68 −0.86) (Table 2). The estimated specificity was 89% (CI 0.08 – 0.15). The estimated positive predictive value was 71% (CI 0.61 – 0.80) and the estimated negative predictive value was 91%, (CI 0.06 – 0.13).

**Table 2:** Performance of the HeartLogic^TM^ algorithm.

	**Population mean**	**95% confidence interval**
Sensitivity	0.79	0.68 – 0.86
Specificity	0.88	0.08 – 0.15
Positive predictive value	0.71	0.61 – 0.80
Negative predictive value	0.91	0.06 – 0.13

### HeartLogic^TM^ Alert Characteristics

**Figure 3:**
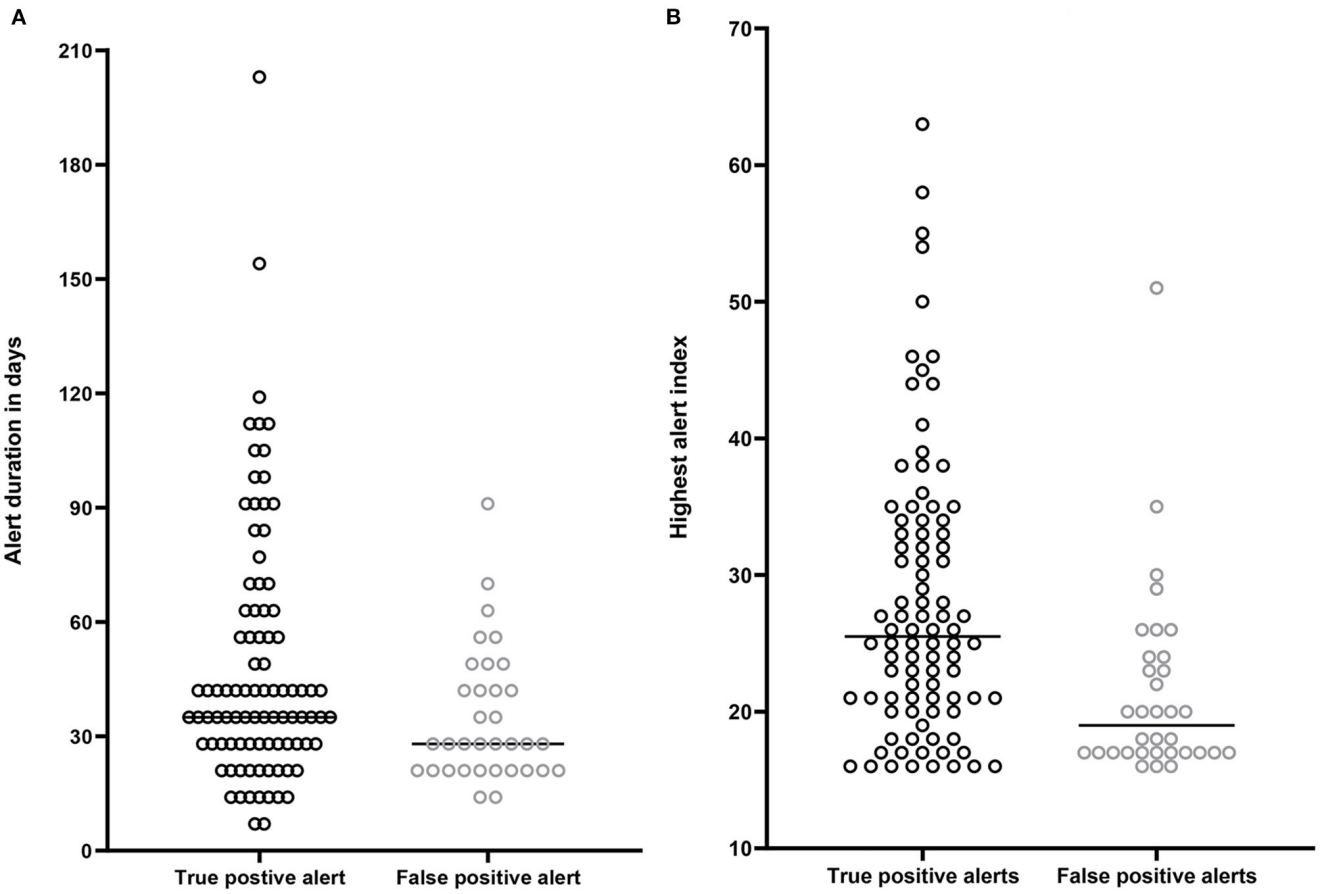
**(A)** Time spend in alert status in days. Median time spend in alert by patients with a true positive alert 42 days [IQR 28–63]. Median time spend in alert by patients with a false positive alert 27 days [IQR 21-44]. **(B)** Highest HeartLogic^TM^ index (from surpassing the alert threshold until below the recovery threshold) in patients with a true positive alert [median 26, (IQR 21–34)] and in patients with a false positive alert [median 19, (IQR 17–24)].

Figure 3A shows the duration of an alert episodes for true positive and false positive alerts. True positive alerts had a median duration of 42 days [IQR 28–63], whereas false positive alerts lasted only 28 days [IQR 21–44]. Lineair mixed models demonstrated a significant estimated effect of −13.9 days (CI−25.9 – −1.9, *p* = 0.02) for false positive alerts compared to true positive alerts. Figure 3B displays the maximal height of the HeartLogic^TM^ index during an alert episode for true positive and false positive alerts. In true positive alert episodes, the maximal HeartLogic^TM^ index value was 26 [IQR 21–34], while in false positive alert episodes the maximal HeartLogic^TM^ index value was 19 [IQR 17–24]. Linear mixed models demonstrated a significant estimated effect of the index value of −5.6 (CI−9.4 – −1.9, *p* < 0.01) for false positive alerts compared to true positive alerts.

### HeartLogic^TM^ Index Height and Severity of Congestion

**Figure 4:**
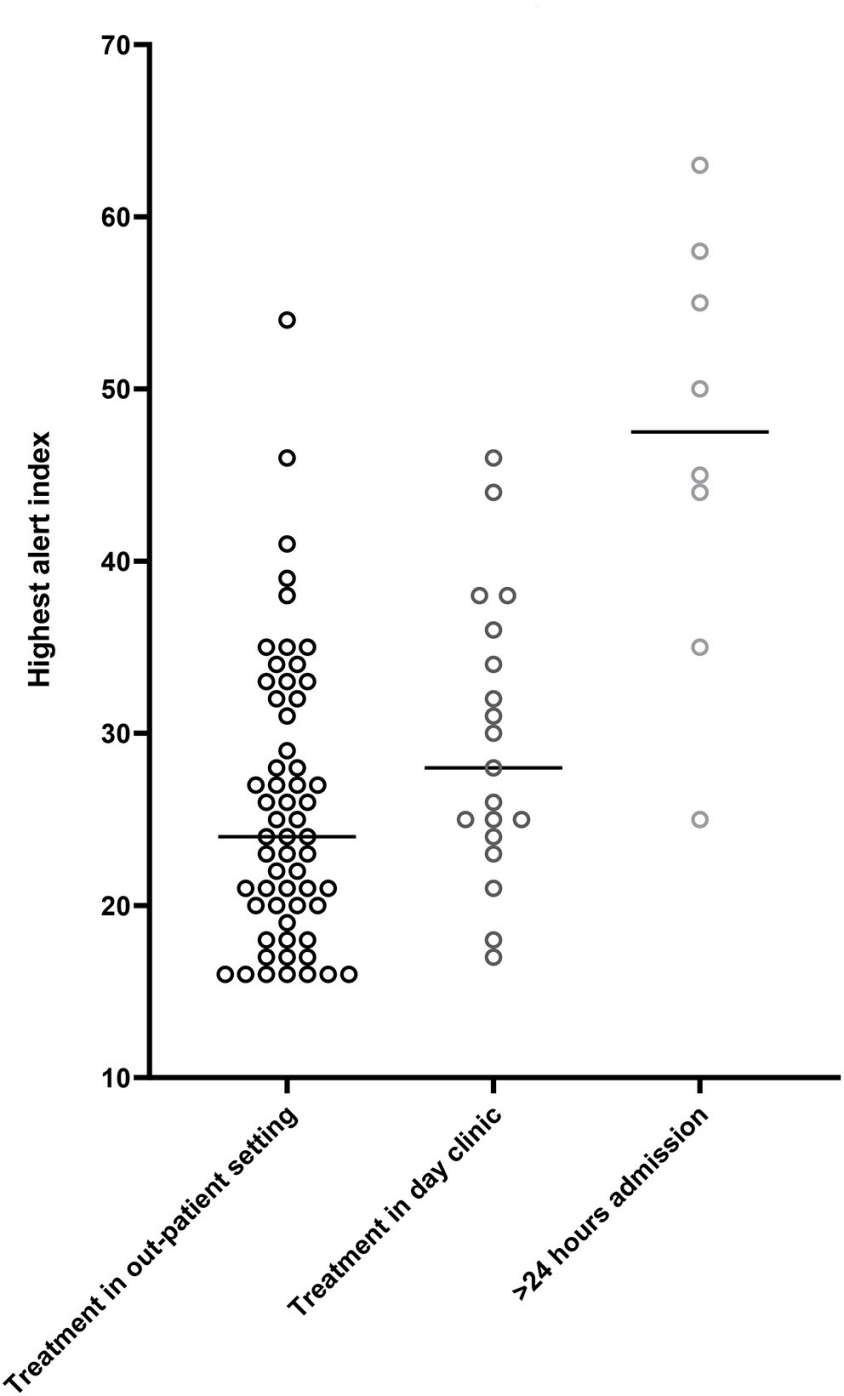
Median highest HeartLogic^TM^ index (from surpassing the alert threshold until below the recovery threshold). Alert height in patients receiving care in the outpatient setting, median 25 [IQR 20–31]. Alert height in patients receiving treatment in the day clinic, median 28 [IQR 24–36]. Alert height of patients receiving >24 h in hospital care, median 45 [IQR 35–58].

During the study period, a primary heart failure related HeartLogic^TM^ alert occurred 85 times. The severity of fluid retention determined the type of treatment. In 57 out of these 85 alerts (67%), the treatment consisted of reinforcement of lifestyle advises and/or optimization of the oral heart failure medication. Of interest, in 8 cases additional optimization of chronic oral heart failure medication was performed next to the temporary escalation in diuretics. In 3 cases a beta-blocker was started after the patient was recompensated, 2 patients started with an ARNI, in 2 cases the MRA was (re-) introduced and 1 patient was started on an ACE-inhibitor. In 20 (24%) alert episodes, administration of intravenous diuretics during a day visit (<24 h) was sufficient, while in the remaining 8 (9%) alert episodes a hospitalization lasting more than 24 h for decompensated heart failure was required. In patients treated in the outpatient setting, the maximal height of the HeartLogic^TM^ index during an alert episode was 25 [IQR 20–31]. In patients intravenously treated in a day visit the maximal HL index was 28 [IQR 24–36] and in patients in whom hospitalization was required the maximal HeartLogic^TM^ index was 45 [IQR 35–58] (Figure 4). Linear mixed models demonstrated that treatment had a significant effect on the height of the HeartLogic^TM^ index. The estimated effect on the HeartLogic^TM^ index of patients treated with intravenous medication was −15.7 (CI−22.8 – −8.7, *p* < 0.01) compared to hospitalized patients. Patients treated in the outpatient setting had an estimated effect of −21.2 (CI−27.6 – −14.7, *p* < 0.01) HeartLogic^TM^ index compared with hospitalized patients.

## Discussion

The main finding of the current study is that the HeartLogic^TM^ algorithm is robust in detecting impending fluid retention in a timely manner in real-world clinical practice setting. The algorithm correctly alerts for upcoming fluid retention in the majority of cases (*n* = 92, 71%), with a rather low unexplained alert rate of 0.23 per patient year. Specifically, the algorithm has a sensitivity of 79% and a specificity of 88% to detect impending fluid retention. The negative predictive value was 91% and the false negative rate was very low with 0.17 events per patient year. Alert height and alert duration seem to be indicative for true positive alerts and, importantly, alert height is correlated with the severity of the fluid retention.

Early detection of congestion is the mainstay of preventing heart failure related hospitalizations. However, in clinical practice, timely recognition remains challenging since symptoms typically occur relatively late (16). Implantable technologies have been developed to detect the early changes associated with subclinical congestion. Previously, CIED-based single sensor impedance measurements were not robust enough to detect impending fluid retention (17–19). Overcoming many of the drawbacks of its predecessors, the HeartLogic^TM^ algorithm combines five pathophysiological CIED-based sensors: the first and the third heart sounds (S1 and S3, respectively) and the ratio of S3/S1, respiration rate, intrathoracic impedance, night heart rate and physical activity to detect impending fluid retention (8, 9). A study from Calò et al. confirmed that CIED measured heart sounds can accurately detect systolic and diastolic left ventricular functional impairment (20). Gardner et al. demonstrated that all individual sensor values of the HeartLogic^TM^ algorithm deviated significantly prior to an event of decompensated heart failure (13).

So far, data on the performance of the HeartLogic^TM^ algorithm in a real-world setting remain limited and analysis on the alert height and duration have not been performed. The landmark trial, entitled MultiSENSE, demonstrated a 70% sensitivity to detect early signs of fluid retention (8). A previous multicenter study comprising 68 patients, partly from our center, reported a sensitivity of 90% (15). The current larger study, covering 148 patient years, showed a sensitivity of 79%. This discrepancy can, at least partly, be explained by methodological differences in the approach used to determine the sensitivity. Previous studies did not account for the dependent risk of recurrent episodes of fluid retention in the same patient and used a simplified “2 by 2” table to evaluate the performance of the algorithm. The current analysis takes the phenomenon of “repeated measures” into account when determining the performance of the HeartLogic^TM^ algorithm. The sensitivity of 79% therefore seems to better reflect the real-world daily practice. In previous studies, the specificity of the HeartLogic^TM^ algorithm to detect impending fluid retention was reported to be 86% in the MultiSENSE and 89% in the prospective analysis from Treskes et al. which is in line with the 88% in the current analysis (8, 15).

In previous studies, the positive predictive value of HeartLogic^TM^ was relatively low: 11% in MultiSENSE and 58% in a study by Capucci et al. that comprised 58 patients (8, 11). Interestingly, the positive predictive value in the current study was strikingly higher with 71%. Several mechanisms may have contributed to the variation in reported positive predictive values. At first, there is no uniform definition of worsening heart failure. In MultiSENSE, worsening heart failure was defined as an episode of congestion requiring intravenous medication and/or hospitalization for at least 24 h. Therefore, episodes that could be managed in the outpatient setting were not taken into account. In the current study, as well as in the study by Capucci et al., these episodes were taken into account and comprised a substantial percentage of the heart failure episodes. At second, while in MultiSENSE the physicians were blinded to the HeartLogic^TM^ index, physicians in the current study were aware of the index. Conceptually, an active alert could have triggered more thorough history taking and follow-up in which mild symptoms could be identified earlier on. At third, the current study comprised a structured heart failure care path after an alert was issued. Patients were followed-up according to a structured pre-defined protocol, which might have identified more cases of mild symptoms.

In the current study, the negative predictive value was 91%, as compared to 99.8% in MultiSENSE (8). Accordingly, both studies evidence that patients who are not in alert status are correctly identified by the algorithm as being at low risk for impending fluid retention. This is substantiated by the current low false negative event rate (0.17 per patient year) which is congruent with the previously reported false negative rate of 0.03 per patient year by Calò et al. (10). The high negative predictive value and low false negative event rate make the HeartLogic^TM^ algorithm very practical for clinical implementation, especially during the COVID-19 pandemic (21, 22). In patients who are not in alert status, the outpatient visits may be converted to digital consults and/or lowered in frequency which is less burdensome for patients while they can rely on the fact that they are continuously monitored. Furthermore, it may save costs and it enables healthcare providers to focus their attention on patients at highest risk of congestion.

To identify patients at highest risk of fluid retention, an analysis of the HeartLogic^TM^ alert characteristics was performed. A previous analysis by Santini et al. showed that alerts were generally shorter and the index was lower, if signs and symptoms of heart failure were treated at an early stage (14). However, to date it remained to be investigated whether the height of the index and the duration of the alert state are indicative of the degree of fluid retention. In the current study, true positive alerts lasted longer than false positive alerts indicating that extra attention is warranted for patients with a persistent in alert status. Furthermore, true positive alerts had a higher maximal HeartLogic^TM^ index compared to false positive alerts and the highest indexes were observed in patients who required hospitalization. Accordingly, the current study shows that alert height is a parameter of interest and justifies physicians to intensify monitoring in patients with a persistently rising HeartLogic^TM^ index.

## Limitations

When interpreting the results of the current study, several factors should be taken into account. First, this was a relatively small single center open label analysis in which the clinicians and the patients were aware of the alert status, which might have introduced bias. Second, this study was conducted during the COVID-19 pandemic, which could have altered patients' behavior and influenced therapy and “contactless care” adherence. However, the pandemic also empowered digital care and thereby guaranteed continuity of care, which is especially valuable in high-risk patients. Third, the study was performed in a tertiary heart failure center with a dedicated heart failure care team with previous experience with digital care and telemonitoring. It remains to be investigated whether the current results are also applicable to hospitals with less experience in this field.

## Future Perspectives

Several studies have validated the HeartLogic^TM^ algorithm for detection of impending fluid retention and have illustrated its potential in the clinical setting (8, 10–15). The algorithm behind the HeartLogic^TM^ index and its weighting of the individual sensor data is not revealed to the users and remains an area of interest. Despite the superiority of the cumulative HeartLogic^TM^ index in predicting clinical events, understanding the predictive values of individual sensors is of importance to guide clinical decision making and facilitate early treatment of patients deemed at highest risk of decompensation. Furthermore, the effectiveness of a HeartLogic^TM^ supported clinical care path might vary depending on individual demographic (e.g. age, gender) and clinical (e.g. etiology of heart failure, left ventricular ejection fraction, intrinsic AV-conduction) characteristics. This should be specifically investigated to redirect health care resources to the patients that are expected to benefit from the HeartLogic^TM^ algorithm most.

## Conclusion

The CIED-based HeartLogic^TM^ algorithm facilitates early detection of impending fluid retention and thereby enables clinical action to prevent this at an early stage. Furthermore, with great certainty, the algorithm identifies patients at low risk of worsening heart failure. Accordingly, resources can be redirected to high-risk patients. The current analysis illustrates that higher and persistent alerts are indicative for true positive alerts and higher index values are indicative for severe fluid retention. These characteristics should be taken into account when implementing HeartLogic^TM^ based follow-up strategies.

## Data Availability Statement

The original contributions presented in the study are included in the article/Supplementary Material, further inquiries can be directed to the corresponding author/s.

## Ethics Statement

The studies involving human participants were reviewed and approved by METC LDD. Written informed consent for participation was not required for this study in accordance with the national legislation and the institutional requirements.

## Author Contributions

AE and SB: study conception and design. MF and RT: data collection. MF and BM: analysis of results and interpretation of results. MF, AE, and SB: interpretation of results and draft manuscript preparation. RT, BM, JJ, and MS: reviewed the results. All authors approved the final version of the manuscript.

## Funding

This work was funded by the general funds of the Department of Cardiology of the Leiden University Medical Center, Leiden, Netherlands. The Department of Cardiology reports receiving unrestricted research and educational grants from Boston Scientific Corporation, Medtronic, and Biotronik. The funders were not involved in the study design, collection, analysis, interpretation of data, the writing of this article, or the decision to submit it for publication.

## Conflict of Interest

AE is a local sub-investigator for the PREEMPT-HF study. RT, SB, and AE received a speaker's honorarium from Boston Scientific in the past 5 years. The Department of Cardiology reports receiving unrestricted research and educational grants from Boston Scientific Corporation, Medtronic, and Biotronik.

The remaining authors declare that the research was conducted in the absence of any commercial or financial relationships that could be construed as a potential conflict of interest.

## Publisher's Note

All claims expressed in this article are solely those of the authors and do not necessarily represent those of their affiliated organizations, or those of the publisher, the editors and the reviewers. Any product that may be evaluated in this article, or claim that may be made by its manufacturer, is not guaranteed or endorsed by the publisher.
