# Supplementary material for: Performance of a HeartLogicTM Based Care Path in the Management of a Real-World Chronic Heart Failure Population
Source: Front Cardiovasc Med. 2022 May 6;9:883873. doi: 10.3389/fcvm.2022.883873 (PMC9120607; doi:10.3389/fcvm.2022.883873)
Supplement: Supplementary file 1 [file Image_1.pdf]

# *Supplementary Material*

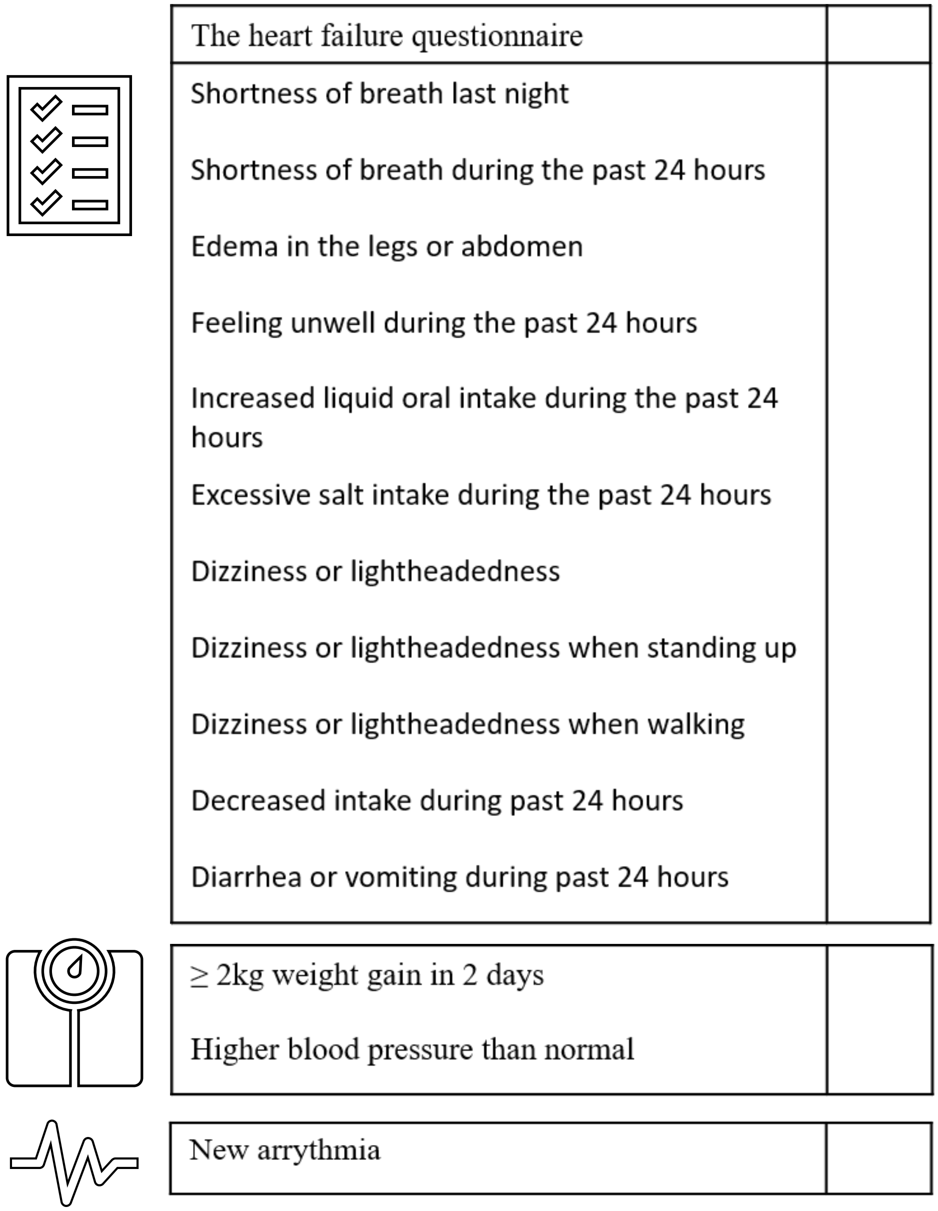

| The heart failure questionnaire | |
|---|---|
| Shortness of breath last night | |
| Shortness of breath during the past 24 hours | |
| Edema in the legs or abdomen | |
| Feeling unwell during the past 24 hours | |
| Increased liquid oral intake during the past 24 hours | |
| Excessive salt intake during the past 24 hours | |
| Dizziness or lightheadedness | |
| Dizziness or lightheadedness when standing up | |
| Dizziness or lightheadedness when walking | |
| Decreased intake during past 24 hours | |
| Diarrhea or vomiting during past 24 hours | |
| ≥ 2kg weight gain in 2 days | |
| Higher blood pressure than normal | |
| New arrythmia | |

**Supplementary Figure 1.** The heart failure questionnaire
